# Characterization of a bifidobacterial system that utilizes galacto-oligosaccharides

**DOI:** 10.1099/mic.0.000100

**Published:** 2015-07

**Authors:** Akira Shigehisa, Hidetsugu Sotoya, Takashi Sato, Taeko Hara, Hoshitaka Matsumoto, Takahiro Matsuki

**Affiliations:** Yakult Central Institute, 5-11 Izumi, Kunitachi, Tokyo 186-8650, Japan

## Abstract

The galacto-oligosaccharide (GOS) OLIGOMATE 55N (Yakult) is a mixture of oligosaccharides, the main component of which is 4′-galactosyllactose (4′-GL). Numerous reports have shown that GOSs are non-digestible, reach the colon and selectively stimulate the growth of bifidobacteria. The product has been used as a food ingredient and its applications have expanded rapidly. However, the bifidobacterial glycoside hydrolases and transporters responsible for utilizing GOSs have not been characterized sufficiently. In this study, we aimed to identify and characterize genes responsible for metabolizing 4′-GL in *Bifidobacterium breve* strain Yakult. We attempted to identify *B. breve* Yakult genes induced by 4′-GL using transcriptional profiling during growth in basal medium containing 4′-GL with a custom microarray. We found that BbrY_0420, which encodes solute-binding protein (SBP), and BbrY_0422, which encodes β-galactosidase, were markedly upregulated relative to that during growth in basal medium containing lactose. Investigation of the substrate specificity of recombinant BbrY_0420 protein using surface plasmon resonance showed that BbrY_0420 protein bound to 4′-GL, but not to 3′-GL and 6′-GL, structural isomers of 4′-GL. Additionally, BbrY_0420 had a strong affinity for 4-galactobiose (4-GB), suggesting that this SBP recognized the non-reducing terminal structure of 4′-GL. Incubation of purified recombinant BbrY_0422 protein with 4′-GL, 3′-GL, 6′-GL and 4-GB revealed that the protein efficiently hydrolysed 4′-GL and 4-GB, but did not digest 3′-GL, 6′-GL or lactose, suggesting that BbrY_0422 digested the bond within Gal1,4-β-Gal. Thus, BbrY_0420 (SBP) and BbrY_0422 (β-galactosidase) had identical, strict substrate specificity, suggesting that they were coupled by co-induction to facilitate the transportation and hydrolysis of 4′-GL.

## Introduction

The gut microbiota plays an important role in human health by providing a barrier for colonization of pathogens, exerting important metabolic functions (fermentation of non-digestible fibres, salvaging energy as short-chain fatty acids, production of vitamins, etc.), and stimulating immune system development. It is increasingly apparent that disruption of the gut microbiota causes many metabolic and immunological dysfunctions. Therefore, scientists have begun to investigate modulation of the gut microbiota using food products or additives, such as probiotics and prebiotics (Fuller, 1989; Gibson & Roberfroid, 1995).

Probiotics, which often include *Lactobacillus* and *Bifidobacterium* spp., are widely used as viable microbial food supplements. Amongst the strains commonly found in probiotics, *Bifidobacterium breve* strain Yakult has been administered as a probiotic to exert beneficial effects on the host organism (Almeida *et al.*, 2012; Kano *et al.*, 2013). Alternatively, a number of clinical studies have suggested that some prebiotics enhance the growth of bifidobacteria. For example, galacto-oligosaccharides (GOSs), which have emerged as major prebiotic oligosaccharides, have been used as food ingredients for at least 30 years and their application has expanded rapidly. GOSs contain a mixture of short-chain oligosaccharides (degree of polymerization 2–6, >95 %), with 4′-galactosyllactose (4′-GL) representing the primary molecular component (Kaneko *et al.*, 2014). Studies have shown that GOSs are selectively consumed by bifidobacteria *in vitro* (Maathuis *et al.*, 2012; Matsumoto *et al.*, 2004; Sharp *et al.*, 2001), leading to increases in the population of bifidobacteria in the adult gut microbiota (Matsumoto *et al.*, 2004; Walton *et al.*, 2012) and the infant gut microbiota (Knol *et al.*, 2005; Sierra *et al.*, 2015).

Recent advances in bacterial genomics have shown that certain *Bifidobacterium* strains (i.e. *Bifidobacterium longum* subsp. *infantis* and *B. bifidum*) possess loci for utilization of human milk oligosaccharides (Garrido *et al.*, 2011; LoCascio *et al.*, 2010; Wada *et al.*, 2008), but the molecular mechanisms through which other *Bifidobacterium* utilize oligosaccharides are largely unknown. However, numerous reports have shown that various strains of bifidobacteria are able to utilize prebiotic GOSs during *in vitro* culture when GOSs are provided as the sole carbon source. The molecular mechanisms mediating the uptake and catabolism of various structural isomers of oligosaccharides are not well characterized.

In this study, we aimed to identify genes responsible for utilization of 4′-GL in *B. breve* Yakult. Our data showed that the substrate specificities of the two proteins upregulated during culture with 4′-GL were the same, suggesting that they were coupled by co-induction to facilitate the transportation and hydrolysis of 4′-GL.

## Methods

### Microarray and quantitative PCR (qPCR) analyses

*B. breve* Yakult was grown in basal medium [10 g peptone l^− 1^, 1 g yeast extract l^− 1^, 3 g KH_2_PO_4_ l^− 1^, 4.8 g K_2_HPO_4_ l^− 1^, 0.2 g (NH_4_)_2_SO_4_.7H_2_O l^− 1^ and 0.5 g l-cysteine/HCl l^− 1^] supplemented with 1 % 4′-GL or lactose under anaerobic conditions at 37 °C. The bacterial cells were collected at the exponential phase, mixed with 2 vols RNAprotect Bacteria Reagent (Qiagen) and harvested by centrifugation. The bacteria were treated with lysozyme (2.5 mg ml^− 1^; Sigma-Aldrich) and *N*-acetylmuramidase (25 μg ml^− 1^; Seikagaku-kogyo) in 10 % sucrose Tris/EDTA (TE) buffer (pH 7.5) at 37 °C for 10 min and RNA was extracted using a Ribo-Pure Bacteria kit (Ambion). Gene expression was investigated using custom microarrays (TaKaRa Bio) designed for *B. breve* Yakult (Ishikawa *et al.*, 2011). The extracted RNAs were labelled with Cy3-dUTP or Cy5-dUTP using an RNA Fluorescence Labelling Core kit (M-MLV Version) Version 2.0 (TaKaRa Bio). The labelled RNAs were mixed and competitively hybridized onto *B. breve* Yakult custom microarrays. Microarrays were then scanned with a DNA Microarray Scanner (Agilent) according to the manufacturer's protocol. The acquired image was analysed by GenePix Pro 4.1 (Molecular Devices). Clustering analysis was performed with Acuity 4.0 (Molecular Devices) and scatter plot analysis was performed with SilicoCyte (SilicoCyte). For reverse transcription (RT)-qPCR analysis, complementary DNA was synthesized from RNA with a PrimeScript 1st strand cDNA synthesis kit (TaKaRa Bio), and real-time PCR was performed with an ABI Prism 7500 (Applied Biosystems). Each reaction mixture (20 μl) included 1 ×  SYBR Green Master Mix (TaKaRa Bio), 0.25 μM specific primers and template cDNA. The following specific primers were designed and/or used in this study: 5′-GGCGAGAACATCACTATCAACATG-3′ and 5′-GATGAGCTTCTGGACGAATTGG-3′ for BbrY_0420, 5′-CACCCGCTTCGATTTCTACCT-3′ and 5′-GCCTTCCACACCGTTGATG-3′ for BbrY_0422, and 5′-TCAACTTCGGCGCTTTCG-3′ and 5′-GACAGCTCGGAAACGTGGAT-3′ for BbrY_0788 (ribosomal S1 protein) (Ishikawa *et al.*, 2011). The amplification program consisted of one cycle of 95 °C for 10 min, 45 cycles of 95 °C for 5 s and 60 °C for 36 s, and one cycle of 94 °C for 15 s. Melt curve analysis was performed after amplification to distinguish the targeted PCR product from the non-targeted PCR product. Quantification values were expressed as the change relative to the lactose culture used as the reference. The relative expression of each gene was normalized to that of a putative housekeeping gene encoding ribosomal S1 protein (BbrY_0788).

### Preparation of recombinant protein

BbrY_0420 and BbrY_0422 proteins were produced by recombination with an N-terminal His_6_-tag. The strains were cultured in GAM broth (Nissui) supplemented with 1 % glucose and 1 % lactose, collected by centrifugation, treated with 2.5 mg lysozyme ml^− 1^ and 25 μg *N*-acetylmuramidase ml^− 1^ in 10 % sucrose TE buffer (pH 7.5) at 37 °C for 10 min, and the genomic DNA was extracted by a MagExtractor-Genome- (Toyobo). Target genes were amplified by PCR using PrimeSTAR Max polymerase (TaKaRa Bio) with the following primers: BbrY4065-420NdeIFw (5′-GCCGCATATGCGCAACACCAAGAAGGTCATCGCGGCA-3′, where the underline indicates the *Nde*I site), BbrY4065-420XhoIRv (5′-GCCGCTCGAGTCACTTGACGGTGACGTTGTAGCCCTGCTG-3′, where the underline indicates the *Xba*I site), BbrY4065-BbrY_0422NdeIFw (5′-GCCGCATATGATGACTACTCGTAGAGCATTTAGGTGGCCG-3′, where the underline indicates the *Nde*I site) and BbrY4065-BbrY_0422XbaIRv (5′-GCCGTCTAGATTAGCAGGACGTTTTAGCGATAAGAATGGCGTT-3′, where the underline indi cates the *Xba*I site). The amplified fragments were inspected by gel electrophoresis, purified using a GeneClean kit (MP Biomedicals) and inserted into the pCold I vector (TaKaRa Bio). After confirmation of the nucleotide sequences, the plasmids were introduced into *Escherichia coli* BL21 (TaKaRa Bio).

Recombinant protein expression and purification were performed using a pCold DNA Cold Shock Expression System (TaKaRa Bio) according to the manufacturer's instructions. Briefly, *E. coli* BL21 harbouring the pCold I vector were cultured in 30 ml LB broth containing 100 μg ampicillin ml^− 1^ at 37 °C. When the OD_600_ reached 0.6–0.8, culture media were incubated at 15 °C for 30 min and IPTG (1.0 mM in final concentration) was added to the medium. The culture media were then incubated at 15 °C for 24 h with shaking to induce overexpression of the inserted gene.

The cells (∼0.5 g) were harvested by centrifugation (20 400 ***g*** at 4 °C for 15 min), suspended in 2 ml B-PER Bacterial Protein Extraction Reagent (Thermo Fisher Scientific) containing 4 μl lysozyme (50 mg ml^− 1^; Sigma-Aldrich) and 4 μl DNase I (5 U μl^− 1^; TaKaRa Bio), and incubated at room temperature for 10 min. The recombinant proteins were purified using a Ni-NTA Spin Column (Qiagen) according to the manufacturer's instructions. Protein concentration was determined spectrophotometrically using a NanoDrop 2000 (Thermo Fisher Scientific).

### Surface plasmon resonance (SPR) binding assays using Biacore technology

The affinity of the recombinant BbrY_0420 protein with various carbohydrates was determined using a Biacore tbl200 (GE Healthcare). The recombinant protein (50 μg ml^− 1^) was immobilized on a CM5 sensor chip using a random amine coupling kit at pH 4.0 (GE Healthcare). Various concentrations of carbohydrates [4′-GL, 3′-GL, 6′-GL, 4-galactobiose (4-GB) and lactose; 0–250 μM] in HBS-P+ buffer (GE Healthcare) were injected at a flow rate of 30 μl min^− 1^ for the association (60 s) and the dissociation (90 s). Experiments were performed in triplicates at 25 °C. The data were analysed with Biacore tbl200 evaluation software (GE Healthcare). Equilibrium dissociation constants (*K*_d_) were calculated by fitting a one-site binding model to either the steady-state response data or the full sensorgrams to measure the binding kinetics.

### Carbohydrates used in this study

**Fig. 1.**
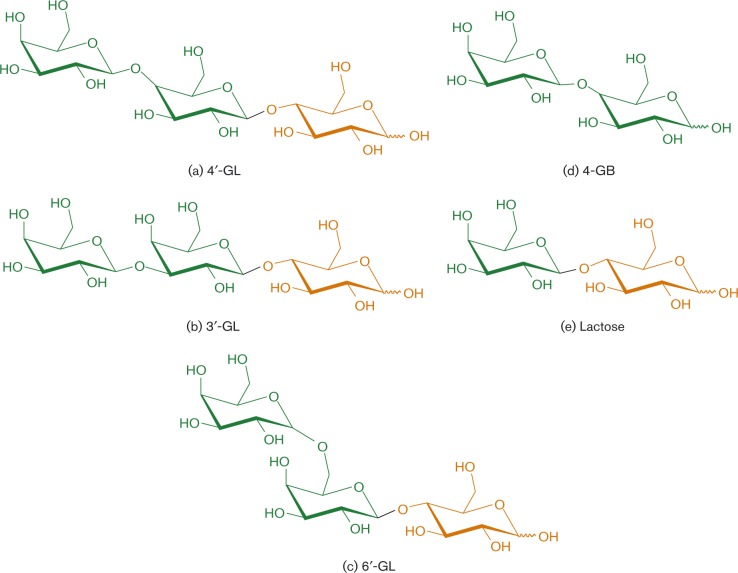
Structure of the carbohydrates used in this study: (a–c) three galactosyllactose isomers, (d) 4-GB and (e) lactose.

4′-GL was kindly provided by Nissin Sugar. 4-GB was purchased from Sigma-Aldrich. 3′-GL and 6′-GL were purchased from Carbosynth. The structures of these compounds are shown in Fig. 1.

### Enzyme assays and substrate specificity analyses

Recombinant BbrY_0422, annotated as β-galactosidase (60 nM), was incubated with 3′-GL, 4′-GL, 6′-GL or 4-GB in 50 mM sodium phosphate (pH 6.5) in a reaction mixture (1 ml) at 30 °C for 90 min. Every 30 min, 200 μl aliquots were collected, and the carbohydrate profile was analysed by HPLC (Prominence System; Shimadzu) with an L-column2 ODS (Chemicals Evaluation and Research Institute) according to previously described methods (Matsuura & Imaoka, 1988).

### BLAST analysis of BbrY_0420 and BbrY_0422

Sequence homologues of BbrY_0420 and BbrY_0422 were searched against the National Center for Biotechnology Information protein database restricted to genus *Bifidobacterium* (taxonomy ID: 1678) using blast (Altschul *et al.*, 1997) with parameters set as follows: *E* value < 10, identity >60 % and query coverage >60 %. The sequences assigned by bidirectional best hit with BbrY_0420 or BbrY_0422 were aligned with ClustalX2.1 (Larkin *et al.*, 2007). The resulting phylogenetic tree file was visualized using FigTree version 1.4.0 (http://tree.bio.ed.ac.uk/software/figtree). The sequences of BbrY_0420 and BbrY_0422 were deposited in the DDBJ database under accession numbers LC015362 and LC015363, respectively.

## Results

### Upregulation of genes in *B. breve* Yakult during growth with 4′-GL

**Fig. 2.**
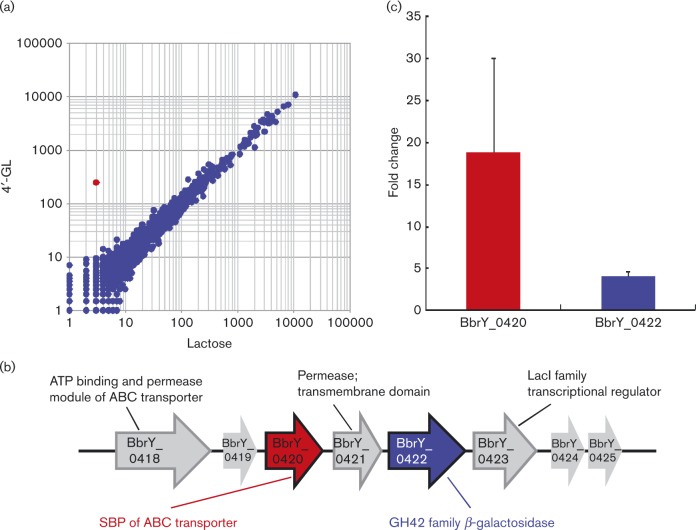
(a) Representative scatter plots of pairwise comparisons of the oligosaccharide-induced differential global transcriptome in *B. breve* Yakult. All genes are shown by solid blue circles except for BbrY_0420, which is shown in red. (b) Organization of the putative 4′-GL utilization locus in *B. breve* Yakult. Genes are listed with locus tag numbers and annotated gene functions are indicated. (c) Fold changes in the expression of BbrY_0420 and BbrY_0422 during 4′-GL culture compared with lactose culture, as evaluated by RT-qPCR. Data represent mean ± sd of three technical replicates.

To identify genes that were responsible for utilization of 4′-GL, *B. breve* Yakult was cultured with 4′-GL or lactose as the only carbon source and gene expression profiles were compared using custom microarrays. As shown in Fig. 2(a), BbrY_0420, which encodes SBP of the ATP-binding cassette (ABC) transporter (Fig. 2b), was markedly upregulated during growth in 4′-GL compared with that in lactose, suggesting the involvement of this protein in 4′-GL utilization. Additionally, BbrY_0422, which encodes β-galactosidase and belongs to the glycoside hydrolase GH42 gene family, was upregulated 3.5-fold compared with that in lactose. qPCR analysis of BbrY_0420 and BbrY_0422 confirmed that these targets were upregulated by 19 ± 11- and 4.0 ± 0.6-fold (mean ± sd), respectively, during growth in 4′-GL (Fig. 2c).

### Preparation of recombinant BbrY_0420 and BbrY_0422 proteins

The genes encoding SBP (BbrY_0420) and β-galactosidase (BbrY_0422) were cloned into an expression vector to produce the recombinants in *E. coli* BL21. From 30 ml LB broth, we obtained 1.44 and 0.77 mg recombinant BbrY_0420 and BbrY_0422 protein, respectively (Fig. S1, available in the online Supplementary Material).

### Substrate specificity of BbrY_0420 as assessed by SPR

**Fig. 3.**
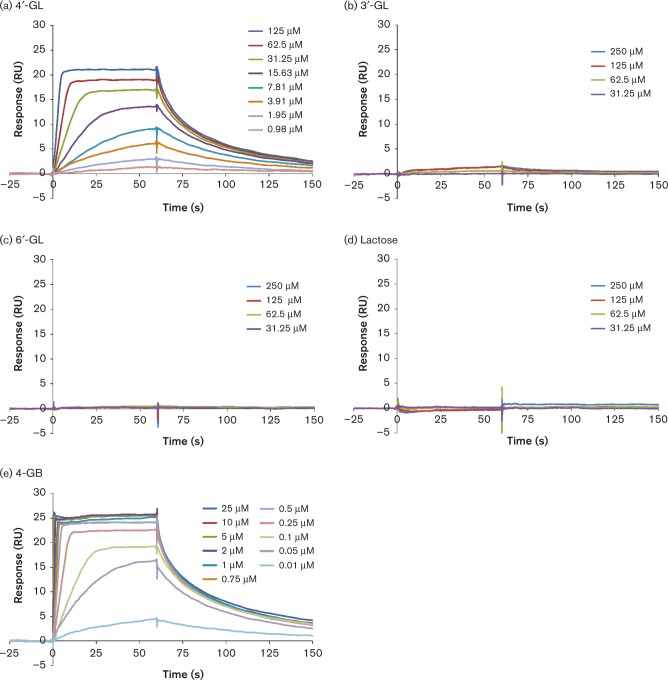
Overlay plot of SPR sensorgrams showing the association between BbrY_0420 (SBP of the ABC transporter) and various carbohydrates: (a) 4′-GL, (b) 3′-GL, (c) 6′-GL, (d) lactose and (e) 4-GB. RU, resonance units.

To evaluate the biochemical interactions between BbrY_0420 protein and various carbohydrates, we measured the SPR of the recombinant using a Biacore system. Fig. 3(a) shows the SPR sensorgrams of the protein and various concentrations of 4′-GL. Increasing concentrations of 4′-GL in the solution led to proportional increases in the SPR signal intensity, indicating that SBP bound to 4′-GL in a concentration-dependent manner. The kinetics of ligand binding were calculated and the equilibrium dissociation constant (*K*_d_) was found to be *K*_d_ = 1.04 × 10^–5^ M. In contrast, the SPR sensorgrams for 3′-GL and 6′-GL indicated that SBP did not bind to these structural isomers of 4′-GL and lactose (Fig. 3b–d). We also found that SBP bound to 4-GB and showed a higher *K*_d_ value of 2.37 × 10^− 8^ M (Fig. 3e). These result suggested that SBP recognized the non-reducing terminal structure of 4′-GL (Fig. 1).

### Substrate specificity of the β-galactosidase BbrY_0422 as assessed by degradation activity

**Fig. 4.**
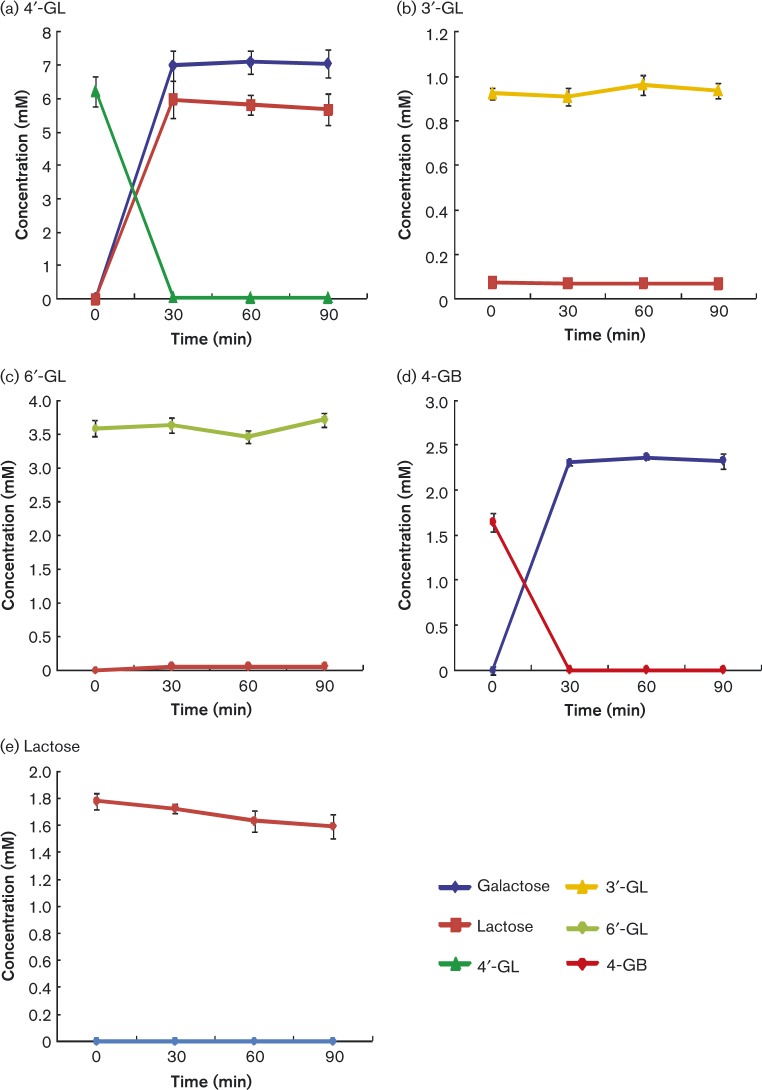
Degradation activity of the recombinant β-galactosidase BbrY_0422: (a) 4′-GL, (b) 3′-GL, (c) 6′-GL, (d) 4-GB and (e) lactose. Data represent mean ± sd of three technical replicates.

To evaluate the specificity of the β-galactosidase, we measured degradation activity with various carbohydrates (i.e. 4′-GL, 3′-GL, 6′-GL, lactose or 4-GB; Fig. 4). The recombinant protein efficiently hydrolysed 4′-GL, and produced equal amounts of galactose and lactose. However, the protein showed almost no activity toward lactose (Gal1,4-β-Glc), suggesting that this β-galactosidase hydrolysed the Gal1,4-β-Gal bond, but not the Gal1,4-β-Glc bond. We also confirmed that 4-GB was hydrolysed by BbrY_0422, whilst the structural isomers (3′-GL and 6′-GL) were not.

### Presence of BbrY_0420 and BbrY_0422 homologues amongst bifidobacteria

To assess the universality of our observations, we performed a blast search for BbrY_0420 and BbrY_0422 against bifidobacterial genes deposited in public databases (Nelson *et al.*, 2010). These analyses showed that homologues of BbrY_0420 were commonly found within *B. breve*, *B. longum*, *B. catenulatum*, *B. pseudocatenulatum* and *B. dentium*, as shown in Fig. S2(a). We also found that some bifidobacteria, such as *B. bifidum* and *B. adolescentis*, had paralogues with higher similarity to the other SBP of *B. breve* Yakult (BbrY_0536, data not shown). However, homologues of BbrY_0422 were more frequently present within bifidobacteria, as shown in Fig. S2(b).

## Discussion

Many reports have shown that GOSs are non-digestible, reach the colon and selectively stimulate the growth of bifidobacteria. However, the molecular mechanisms through which oligosaccharides are utilized are largely unknown. In this study, the transcriptomes of *B. breve* Yakult induced by 4′-GL were analysed to identify the genes involved in the uptake and catabolism of the main components of GOSs. Microarray and RT-qPCR analyses showed that BbrY_0420 (encoding SBP of the ABC transporter) and BbrY_0422 (encoding a β-galactosidase) were upregulated in medium containing 4′-GL as the sole carbon source. These two proteins also had the same substrate specificity. Thus, our study suggested that these adjacent genes may form a gene cluster and are likely co-induced in response to the substrate.

### Gene expression of BbrY_0420

A previous study reported the upregulation of a homologue of BbrY_0420 in response to a mixture of 3′-GL, 4′-GL and 6′-GL using transcriptome analysis (O'Connell Motherway *et al.*, 2011). In our work, we further investigated changes in gene expression using 4′-GL alone; our data confirmed the upregulation of BbrY_0420 with purified 4′-GL, which expanded upon previous studies of this target using both gene expression analysis and a biochemical approach.

### Substrate specificity of the SBP BbrY_0420

In this study, we found that the SBP BbrY_0420 bound to 4′-GL and 4-GB, but not to 3′-GL, 6′-GL or lactose, indicating that BbrY_0420 bound with Gal1,4-β-Gal, the non-reducing terminal structure of 4′-GL. Ejby *et al.* (2013) reported that an SBP from *Bifidobacterium animalis* subsp. *lactis* Bl-04 displays a broad substrate specificity for xylo-oligosaccharides (*BIA*XBP). Parche *et al.* (2007) demonstrated that an SBP from *B. longum* NCC2705 is induced by fructose, ribose and xylose, and binds to these sugars. These data indicate that each type of SBP has its own specificity and recognition site. Therefore, further studies are required to determine the three-dimensional structure of this binding protein in complex with its ligand 4′-GL to elucidate the molecular features of 4′-GL captured by X-ray crystallography or NMR. In addition, the genes/loci that are responsible for 3′-GL and 6′-GL should be further investigated as *B. breve* Yakult utilizes both 3′-GL and 6′-GL (Matsumoto *et al.*, 2004).

Interestingly, BbrY_0420 exhibited higher affinity for 4-GB than 4′-GL. This result may suggest that the primary substrate of BbrY_0420 protein in the gut environment is 4-GB rather than 4′-GL. Sumiyoshi *et al.* (2004) reported that 4′-GL is found in the breast milk of humans and bovines (1.2–1.8 mg l^− 1^), but the concentration in the milk is much lower than major human milk oligosaccharides components, such as fucosyllactose (Sumiyoshi *et al.*, 2004). However, galactobiose is generated as a result of degradation of galactan and could be more abundant in the gut (O'Connell Motherway *et al.*, 2011). Taken together, these results suggest that *B. breve* expresses an SBP that can utilize galactobiose, and that the prebiotic 4′-GL is transported into the cell by this SBP and digested by β-galactosidase BbrY_0422, thereby allowing *B. breve* Yakult to use 4′-GL as an energy source.

### Substrate specificity of the GH42 β-galactosidase BbrY_0422

Degradation activity analysis of BbrY_0422 showed that the substrates of BbrY_0422 were 4′-GL and 4-GB. These results were consistent with a report by Viborg and Katayama (2014), who showed that the BbrY_0422 homologue of *B. longum* subsp. *infantis* ATCC 15697 (Bga42B, 68 % amino acid sequence similarity with BbrY_0422) hydrolyses 4-GB and 4′-GL with high efficiency. Thus, the substrate specificities of BbrY_0420 and BbrY_0422 were the same, indicating that the genes encoding these two proteins form a 4′-GL/4-GB utilization locus.

### Gene cluster for utilization of 4′-GL and 4-GB

ABC transporters consist of transmembrane domains, intracellular nucleotide-binding domains and an extracellular SBP, which governs the specificity and high-affinity capture of substrates (Cui & Davidson, 2011). In our study, SBP (BbrY_0420), the ATP-binding domain protein (BbrY_0418) and permease (BbrY_0421) of the ABC transporter were located in close proximity in the genome. In addition, GH42 β-galactosidase (BbrY_0422), which had the same substrate specificity as BbrY_0420, was present downstream of these ABC transporter components. Thus, our data suggested that these genes made a gene cluster, which exhibited coupled regulation and mediated the transport and hydrolysis of 4′-GL and 4-GB. This gene cluster may also include BbrY_0423, a LacI family transcriptional regulator. Therefore, further studies should investigate the involvement of BbrY_0423 with this gene cluster (Fig. 2b).

### Gene clusters for utilization of 3′-GL and 6′-GL

The results of this study demonstrated that 3′-GL and 6′-GL were not substrates of BbrY_0420 and BbrY_0422 proteins. However, *B. breve* Yakult was able to utilize 3′-GL and 6′-GL (data not shown), indicating that there may be other genes responsible for utilizing these structural isomers. As 3′-GL and 6′-GL are present as subcomponents of OLIGOMATE 55N (Yakult) or as main components of some commercial GOS products (Kaneko *et al.*, 2014), further studies are needed to investigate the genes responsible for utilizing these structural isomers.

## Conclusion

This study provided novel insights into the molecular features responsible for the utilization of 4′-GL, the main GOS prebiotic and a known bifidogenic agent. Determination of the carbohydrate assimilation system of bifidobacteria will facilitate our understanding of how these species are able to adapt to their ecological niche (Sangwan *et al.*, 2011). The identification of prebiotic oligosaccharide utilization loci for probiotic *Bifidobacterium* will also provide useful information for selecting the combination of probiotics for synbiotic therapy.
